# Clinical Utility of Plasma Lipid Peroxidation Biomarkers in Alzheimer’s Disease Differential Diagnosis

**DOI:** 10.3390/antiox9080649

**Published:** 2020-07-22

**Authors:** Carmen Peña-Bautista, Lourdes Álvarez, Thierry Durand, Claire Vigor, Ana Cuevas, Miguel Baquero, Máximo Vento, David Hervás, Consuelo Cháfer-Pericás

**Affiliations:** 1 Neonatal Research Unit, Health Research Institute La Fe, 46026 Valencia, Spain; carpebau@alumni.uv.es (C.P.-B.); maximo.vento@uv.es (M.V.); 2 Neurology Unit, University and Polytechnic Hospital La Fe, 46026 Valencia, Spain; lourdes.alvsa@gmail.com (L.A.); anais85@gmail.com (A.C.); miquelbaquero@gmail.com (M.B.); 3 Institut des Biomolécules Max Mousseron, IBMM, University of Montpellier, CNRS ENSCM, 34093 Montpellier, France; thierry.durand@umontpellier.fr (T.D.); claire.vigor@umontpellier.fr (C.V.); 4 Biostatistical Unit, Health Research Institute La Fe, 46026 Valencia, Spain; bioestadistica@iislafe.es

**Keywords:** plasma, lipid peroxidation, Alzheimer’s disease, differential diagnosis, clinical validation

## Abstract

Background: Differential diagnosis of Alzheimer’s disease (AD) is a complex task due to the clinical similarity among neurodegenerative diseases. Previous studies showed the role of lipid peroxidation in early AD development. However, the clinical validation of potential specific biomarkers in minimally invasive samples constitutes a great challenge in early AD diagnosis. Methods: Plasma samples from participants classified into AD (*n* = 138), non-AD (including MCI and other dementias not due to AD) (*n* = 70) and healthy (*n* = 50) were analysed. Lipid peroxidation compounds (isoprostanes, isofurans, neuroprostanes, neurofurans) were determined by ultra-performance liquid chromatography coupled with tandem mass spectrometry. Statistical analysis for biomarkers’ clinical validation was based on Elastic Net. Results: A two-step diagnosis model was developed from plasma lipid peroxidation products to diagnose early AD specifically, and a bootstrap validated AUC of 0.74 was obtained. Conclusion: A promising AD differential diagnosis model was developed. It was clinically validated as a screening test. However, further external validation is required before clinical application.

## 1. Introduction

Alzheimer’s disease (AD) is the dementia type with the highest incidence worldwide [1]. Its physiopathology is incompletely known and, although it has some specific features, it shares common clinical aspects and metabolic pathways with other neurodegenerative disorders [2]. So, finding specific AD biochemical features is not an easy task. The available therapeutic methods only achieve remarkable symptomatic relief when applied at an early stage. Therefore, clinical validation of potential, early, and specific AD biomarkers in minimally invasive samples is crucial to improve the disease prognosis.

Currently, standard specific AD diagnosis is based on the determination of protein peptides (β-amyloid42, tau), by immunoassay (ELISA technique), in invasively obtained cerebrospinal fluid sampling (CSF) and the expensive brain amyloid PET exams [3]. Recent research has focused on the identification of potential early biomarkers in minimally invasive samples. In general, these new methods show low AD specificity, and they have not been clinically validated [4,5,6]. In fact, a previous study in plasma samples showed high capacity discriminating between AD and healthy participants, but it did not evaluate other similar pathologies [4]. Moreover, few studies have focused on the preclinical AD stage, asymptomatic step detected from CSF biomarkers. For instance, Eruysal et al. discriminated between preclinical AD and healthy participants [5]. In the AD mild cognitive impairment (MCI) stage, patients show cognitive impairment not altering their daily activities, while in mild dementia stage they show an inability to lead a normal life [7]. In this sense, Gao et al. demonstrated that a sensible detection of amyloid 42 peptide is able to differentiate between AD, MCI and healthy participants [8]. Inflammatory biomarkers also could differentiate between AD, MCI and healthy controls [9]. However, other pathologies were not assessed in order to establish the specificity in AD diagnosis.

The main AD histological hallmarks are extracellular senile plaques and neurofibrillary tangles [10]. The former is originated by the extracellular deposition of the accumulated amyloid-beta peptide (i.e., forty-two amino acid long amyloid-beta peptide [Aβ42]). At the same time, the latter is a consequence of intracellular accumulation of tau protein hyperphosphorylated [11]. In fact, it should be reported that cerebrospinal fluid (CSF) concentrations of Aβ42, total tau (t-tau), and hyperphosphorylated tau (p-tau) proteins have been validated as “core” biomarkers of AD pathophysiology. They are pathophysiological biomarkers of amyloid pathology, cortical axonal degeneration, and tangle pathology, respectively [12,13]. In addition, other mechanisms as inflammation or oxidative stress have been related to AD [14]. Specifically, previous studies have shown that lipid peroxidation is involved in the development of neurodegeneration [15]. In this sense, different products derived from lipid peroxidation (e.g., isoprostanes, thiobarbituric acid-reactive substances, fluorescent lipofuscin-like pigments) have been evaluated in different human samples for early AD diagnosis [16,17,18] and results have reflected the difficulty to develop an AD differential diagnosis with this kind of determinations [4].

Nowadays, substantial research has focused on the development of a specific and reliable biochemical AD diagnosis and significant efforts are currently ongoing aimed to enhancing the landscape of blood-based biomarkers for AD [19]. In this regard, some studies have aimed to a diagnosis looking for specific profiles in AD using a combination of several blood biomarkers [20]. Nevertheless, limited specificity was obtained over other neurodegenerative diseases, such as frontotemporal dementia [21,22], Parkinson’s disease [23], or dementia with Lewy bodies (DLB) [24]. Moreover, most of the studies for differential diagnosis are based on CSF samples [25,26]. On the other hand, few studies have clinically validated the potential biomarkers [27]. However, no satisfactory results have been obtained, so further work is required in this line in order to establish new biomarkers which can be validated and applied to the clinical routine.

Therefore, the aim of this work is to develop an early AD diagnosis model, using minimally invasive samples such as plasma that allow a differential diagnosis from other similar neurological and neurodegenerative diseases with similar clinical symptoms. Moreover, we have carried out an internal validation that shows the potential clinical utility of some lipid peroxidation biomarkers in plasma for differential diagnosis of AD.

## 2. Materials and Methods

### 2.1. Study Design and Participants

Participants were aged between 50 and 75 years, and admitted to the Neurology Unit of the University and Polytechnic Hospital La Fe (Valencia, Spain). They were classified into AD group (*n* = 138), non-AD group (*n* = 70) and the healthy group (*n* = 50). The AD group included patients with MCI-AD and mild dementia due to AD who showed cognitive complaints without daily living activities impairment or with minor daily living activities impairment. The non-AD group included patients with MCI not due to AD, frontotemporal dementia, vascular dementia, or DLB. This classification was carried out according to a protocol described in Table 1 based on neuropsychological evaluation (clinical dementia rating (CDR), Repeatable Battery for the Assessment of Neuropsychological Status-Delayed Memory (RBANS.DM)), CSF biomarkers (ß-amyloid, Tau and phosphorylate Tau (p-Tau)), and neuroimaging (amyloid PET), following the National Institute on Aging-Alzheimer’s Association (NIA-AA) recommendations [3].

Regarding exclusion criteria, patients with a history of structural brain disease (tumor, stroke, etc.), major head trauma, epilepsy, multiple sclerosis and major psychiatric disorders were excluded, as well as patients that were not able to undergo neuropsychological evaluations.

The study protocol (project reference number 2016/0257) was approved by the Ethics Committee (CEIC) from Health Research Institute La Fe (Valencia, Spain). The methods were carried out in accordance with relevant guidelines and regulations, and informed consent from all participants was obtained.

### 2.2. Lipid Peroxidation Componuds

Isoprostanes’ standards were from Cayman Chemical Company (Ann Arbor, Michigan, USA) (15(*R*)-15-F_2t-_IsoP, PGE_2_, 2,3-dinor-iPF2 αIII, 15-keto-15-E_2t_-IsoP, 15-keto-15-F_2t_-IsoP, 15-E_2t_-IsoP, 15-F_2t_-IsoP, 5-F_2t_-IsoP, PGF_2α_, 1a,1b-dihomo-PGF_2α_), or synthesized at the Institute of Biomolecules Max Mousseron (IBMM) (Montpellier, France) by Dr Durand’s team (4(*RS*)-F_4t_-NeuroP, 10-*epi*-10-F_4t_-NeuroP, 14(*RS*)-14-F_4t_-NeuroP, Ent-7(*RS*)-F_2t_-dihomo-IsoP, 17-F_2t_-dihomo-IsoP, 17-*epi*-17-F_2t_-dihomo-IsoP, 17(*RS*)-10-*epi*-SC-Δ^15^-11-dihomo-IsoF, 7(*RS*)-ST-Δ^8^-11-dihomo-IsoF) [28].

### 2.3. Sample Treatment

Blood samples were centrifuged for 10 min at 2000 g and plasma samples were stored at −80 °C until the analysis. Samples were thawed on ice and 5 µL of the internal standard solution (PGF_2α_-D_4_ 2 µmol L^−1^ and D_4_–10-epi-10-F_4t_-NeroP 1.2 µmol L^−1^) were added. Then, a basic hydrolysis with potassium hydroxide and a clean-up step with solid phase extraction (SPE) were carried out. Briefly, SPE was carried out using Strata X-AW cartridges, the procedure consisted on a cartridge conditioning step with methanol and H_2_O, a sample loading, washing steps with ammonium acetate buffer (0.1 mol L^−1^, pH 7) and heptane, and an elution step with 2 × 500 µL CH3OH (5% (*v*/*v*) acetic acid). Then samples were evaporated and reconstituted in 100 µL of H2O (0.01% acetic acid (*v*/*v*)):CH3OH (85:15 *v*/*v*). Finally, samples were injected in a chromatographic system, and they were analyzed by ultra-performance liquid chromatography coupled with tandem mass spectrometry (UPLC-MS/MS) [4].

### 2.4. UPLC-MS/MS

The analytical method consists of ultra-performance liquid chromatography coupled to tandem mass spectrometry (UPLC-MS/MS) described in a previous study [4]. Briefly, a Waters Acquity UPLC-Xevo TQD system (Milford, MA USA) and negative electrospray ionization (ESI) were used. The column employed was an Acquity UPLC BEH C18 (2.1 × 100 MM, 1.7 µm). The mobile phase was (A) water (0.01% *v*/*v* acetic acid) and (B) acetonitrile (0.01% acetic acid) [4]. The analytical method was validated in a previous study [4], showing linearity with confidence intervals of 0.990–0.99. In addition, inter-day and intra-day coefficients of variation were 5–13% and 2–11%, respectively.

### 2.5. Statistical Analysis

Variables distribution was studied using a Kolmogorov–Smirnov test. Data were summarized using median (1st, 3rd quartile) in the case of continuous variables and with relative and absolute frequencies in the case of categorical variables. A two-stage model for Alzheimer’s disease diagnosis was developed by adjusting two nested logistic regression models. The first model was based on the discrimination capacity of the neuropsychological evaluation to differentiate between control and case (including AD and non-AD groups) participants. Specifically, the clinical variables RBANS.DM (Repeatable Battery for the Assessment of Neuropsychological Status. Delayed Memory) and CDR (Clinical Dementia Rating) were used as predictors in this first model. The second model was based on the discrimination capacity of lipid peroxidation products from plasma samples to differentiate between AD and non-AD patients in the case group. Specifically, the potential predictors in this second model were 15(*R*)-15-F_2t_-IsoP, PGE_2_, 2,3-dinor-iPF_2α_-III, 15-keto-15-E_2t_-IsoP, 15-keto-15-F_2t_-IsoP, 15-E_2t_-IsoP, 5-F_2t_-IsoP, 15-F_2t_-IsoP, PGF_2α_, 1a,1b-dihomo-PGF_2α_, 4(*RS*)-F_4t_-NeuroP, 10-*epi*-10-F_4t_-NeuroP, 14(*RS*)-14-F_4t_-NeuroP, Ent-7(*RS*)-F_2t_-dihomo-IsoP, 17-F_2t_-dihomo-IsoP, 17-*epi*-17-F_2t_-dihomo-IsoP, 17(*RS*)-10-*epi*-SC-Δ^15^-11-dihomo-IsoF, 7(*RS*)-ST-Δ^8^-11-dihomo-IsoF, as well as the total parameters IsoP, IsoF and NeuroF. Selection of the final predictors in the model was performed using Elastic Net [29]. Performance of the model was assessed by estimating optimism-corrected AUC using 200 bootstrap replications. All statistical analyses were performed using R (version 3.6) and R packages pROC (version 1.14.0) and brms (version 2.8.0).

**Table 1:** Participants’ classification attending to neuropsychological evaluation, neuroimage and cerebrospinal fluid biomarkers.

Tests	AD Group	Non-AD Group	Healthy Group
Neuropsychological tests			
CDR [30]	0.5–1	0.5–1	0
RBANS.DM [31]	≤85	≤85	>85
Neuroimage tests			
Amyloid PET	Positive	Negative	Negative
CSF biomarkers [32,33]			
β-amyloid (pg mL^−1^)	≤725	≥725	≥725
t-tau (pg mL^−1^)	≥85	≤85	≤85
p-tau (pg mL^−1^)	≥350	≤350	≤350

CDR: Clinical dementia rating; RBANS.DM: Repeatable Battery for the Assessment of Neuropsychological Status-Delayed Memory; CSF: cerebrospinal fluid; t-tau: total tau; p-tau: phosphorylated tau.

## 3. Results

The demographic and clinical data from the participants are summarized in Table 2. The clinical variables allowed to differentiate among participants groups. Specifically, the CSF biomarkers (ß-amyloid42, Tau, p-Tau) levels identify AD patients from control and non-AD participants. Moreover, the neuropsychological evaluation (RBANS.DM, CDR) identifies control participants.

The analytes concentrations found in plasma samples from participants groups are summarized in Table 3. All these variables showed non-normal distribution, so the non-parametric test (Kruskal–Wallis) was applied showing statistically significant differences among groups for some lipid peroxidation compounds (15-E2t-IsoP, PGF2α, 4(RS)-F4t-NeuroP, 10-epi-10-F4t-NeuroP, IsoP).

**Figure 1:**
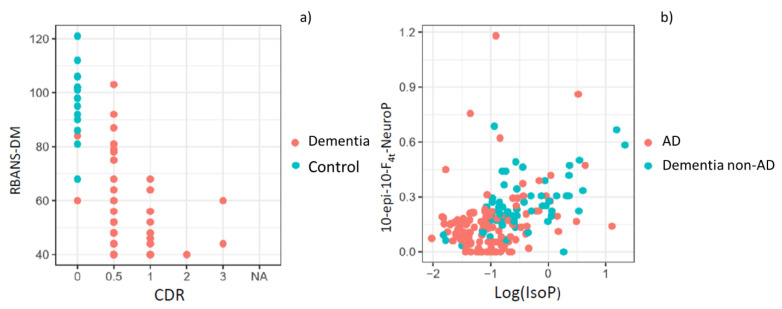
(**a**) Representation of control and dementia patients by using standard neuropsychological evaluation tests (RBANS-DM, CDR); (**b**) Representation of AD and non-AD patients by using the variables 10-*epi*-10-F_4t_-NeuroP and IsoP.

The first model, using these neuropsychological variables, was able to discriminate between control and patients. It achieved a very high accuracy, with an AUC of 0.99 and a bootstrap validated AUC of 0.99. These results show that separating control participants from case patients (AD, non-AD) is straightforward using standard neuropsychological evaluation tests. In Figure 1a, it can be seen that participants without any neurological or neurodegenerative disease (healthy participants) are grouped in the left and upper side, indicating higher RBANS.DM and lower CDR punctuations. The formula for this first prediction step is the following:

(1) $$\mathrm{Pr}\left(Case/Control\right)=\frac{e^{9.25-0.13\times RBANS+22.71\times CDR}}{1+e^{9.25-0.13\times RBANS+22.71\times CDR}}$$

The second model, for discriminating between AD and non-AD patients in the case group included the variables 10-epi-10-F4t-NeuroP and IsoPs (Figure 1b), and it achieved an AUC of 0.79 and a bootstrap validated AUC of 0.74. Calibration of the model was satisfactory. It was assessed using bootstrapping and comparing predicted vs. obtained values, observing very low deviations. The formula for this final prediction step, to be applied only to the individuals predicted as patients (case) by the first step, is the following:

(2) $$\mathrm{Pr}\left(AD/non-AD\right)=\frac{e^{-0.14+1.15\times log\left(IsoP_{s}\right)+2.24\times 10-epi-10-F4t-NeuroP}}{1+e^{-0.14+1.15\times log\left(IsoP_{s}\right)+2.24\times 10-epi-10-F4t-NeuroP}}$$

## 4. Discussion

In this work it is described a new diagnosis model based on plasma lipid peroxidation biomarkers and neuropsychological scores, which evaluate memory, cognition and functional performance. This model could be able to differentiate AD from healthy subjects and participants with other pathologies, such as MCI not due to AD, frontotemporal dementia, vascular dementia, or DLB. Differential diagnosis between AD and non-AD pathologies are commonly a challenge in neurology units especially in early stages [34], since some pathologies show similar clinical symptoms. Therefore, a reliable early diagnosis model is required to be applied to clinical practice.

Recent research has shown an increasing interest in the clinical validation of potential biomarkers to early and specific diagnose AD using minimally invasive biological samples [35]. Among the physiological mechanisms that are already impaired in early disease stages, lipid peroxidation has shown some promising results, and plasma samples constitute an interesting matrix in the search for the corresponding biomarkers [16,36,37,38,39,40,41,42].

Among lipid peroxidation biomarkers evaluated in plasma, some AD studies found altered levels for malondialdehyde [36,37,38,42], 4-hydroxynonenal [39], lipophilic fluorescent products [40,41], and isoprostanes [4]. In general, these potential biomarkers showed elevated levels in AD in comparison with healthy participants, reflecting high oxidative stress at systemic level. However, oxidative stress is common in many pathologies, such as cancer [43] or vascular diseases [44], as well as in other neurodegenerative diseases [45]. For that reason, the present work focused on the need to develop a specific diagnosis model for AD. In fact, AD shows similar clinical symptoms to other pathologies, and the differential AD diagnosis constitutes the real diagnostic challenge. In this sense, lipid peroxidation biomarkers were evaluated as potential specific AD biomarkers, as the brain has a high lipid composition (polyunsaturated fatty acids…) [46]. For this, a previously developed and validated analytical method was applied [4]. This method showed adequate linearity for all the analytes within the corresponding concentration ranges, and suitable precision. The limits of detection and accuracy were satisfactory, and matrix effect was considered negligible. Among studied compounds, statistically significant results were obtained for two prostaglandins (derived from araquidonic acid), two neuroprostanes (derived from docosahexanoic acid), and isoprostanes as total parameter (15-E2t-IsoP, PGF2α, 4(RS)-F4t-NeuroP, 10-epi-10-F4t-NeuroP, IsoP). In contrast to the results in this work, some studies determining isoprostanoids did not obtain satisfactory results [47,48]. It could be explained by the limited list of compounds assessed in literature. However, in the present study a set of 18 compounds were evaluated simultaneously, and it could provide more information about the oxidative state of each individual.

In addition, the present study shows the strengths of using standard diagnosis based on biological definition (CSF biomarkers) to identify accurately the participants (early AD patients, healthy controls, non-AD patients). Furthermore, it is important to highlight the relevant discrimination capacity of the neuropsychological evaluation to identify accurately the healthy controls. From this accurate participant’s classification, a further AD specific and minimally invasive diagnosis was developed. For this, a two-step model was required using the advantages of the neuropsychological evaluation (first step), and the plasma lipid peroxidation determinations (second step). In the developed model, the first step identified the healthy participants, while the second step increased the diagnosis specificity, differentiating AD patients from other patients with other pathologies with similar symptoms. In this sense, a one-step model would not be able to distinguish accurately among AD, non-AD and healthy patients. Therefore, the two-step developed model was required to achieve the minimally invasive and differential AD diagnosis.

Regarding AD differential diagnosis, our study achieved high discriminative power. Albeit not outstanding, it serves as a first approach for developing a differential diagnosis model based on lipid peroxidation compounds. Some studies can be found in literature identifying different biomarkers that differentiate AD from vascular dementia [49], and diabetes-related dementia [50]. However, there is a lack of preliminary studies with clinical validation. A recent study focused on differentiating AD and DLB by means of different pathological signatures of gait [51] supported the theory of interacting cognitive-motor networks [52]. In addition, a previous study found that the CSF p-tau181/Aβ42 ratio might reliably detect AD pathology in patients suffering from different types of dementia [26]. In the present work the non-AD group included a large variety of pathologies, such as MCI not due to AD, frontotemporal dementia, vascular dementia, and DLB. The different lipid peroxidation pattern observed between AD and non-AD subjects could be corroborated by a previous study, which suggested that high lipid peroxidation levels preceded β-amyloid accumulation in brain [53]. Among the physiological mechanisms that could explain the different lipid peroxidation levels between AD and non-AD pathologies, it is important to highlight the role of potential mediators between lipid peroxidation products and AD pathology [54]. Specifically, thromboxane A2 receptor is activated by isoprostanes and promotes amyloid aggregation [55,56]. In fact, previous studies have shown that agonists for this receptor reduced this amyloid increase and they could be potential treatments for AD [55]. On the other hand, another study found co-localization of lipid oxidation and amyloid plaques in brain [57]. From the clinical point of view, the specificity described in the developed diagnosis model could have a great value due to the high clinical similarity among pathological symptoms.

As regards biomarkers and neuropsychological tests, they were selected from our previous experience. In fact, a study carried out with the same lipid peroxidation compounds in plasma samples from AD and healthy participants showed the capacity of these analytes as potential biomarkers for AD [4]. In that work, a one-step diagnosis model was developed from the levels obtained for six lipid peroxidation compounds. The corresponding diagnosis model could differentiate early AD patients from healthy participants with satisfactory accuracy (AUC-ROC 0.817). Nevertheless, it showed the disadvantage of low sample size. Moreover, the differential diagnosis power from non-AD pathologies, which constitutes an important diagnostic problem in clinical practice, was not evaluated [4]. On the other hand, a previous model for early AD diagnosis was developed from the RBANS.DM test. It showed a high discriminative power between AD and non-AD participants [58]. For that reason, RBANS.DM was included in the first step of the present model, improving biomarkers diagnosis power. In this sense, the present developed diagnosis model is based on two steps, the sample size has been suitable to carry out an internal clinical validation, and the differential diagnosis has been included.

Finally, few studies have carried out an external clinical validation of potential biomarkers (plasma proteins, magnetic resonance imaging scans) differentiating two groups of participants (discovery group, validation group) [59,60]. In order to improve the statistical power, other studies developed an internal clinical validation [61,62]. Similarly, in this work, an internal clinical validation was carried out obtaining a satisfactory diagnostic power, since a large sample size was available. Most of previous works were based on CSF biomarkers or neuroimaging biomarkers, so the internal clinical validation based on plasma lipid peroxidation biomarkers constitutes a promising new approach.

The two-step diagnosis model developed in the present work provides the probability of suffering AD from early stages. In the first step, in a given population, it is possible to discriminate the control patients of case patients and thus putative AD patients. In the second step, AD diagnosis can be differentiated from other neurodegenerative diseases also involving cognitive impairment. These results combined with other factors (e.g., age, gender, familiar background, risk factors…) could decide upon the further need of using invasive techniques to establish the patient’s diagnosis [63]. Therefore, the present diagnosis model could be considered a relevant approach in the clinical practice field.

## 5. Conclusions

A two-step early and differential diagnostic model has been developed indicating the individual probability of suffering from early AD, using low cost and minimally invasive procedures for the potential diagnosis. It consisted of a simultaneous approach from neuropsychological and biochemical fields. Lipid peroxidation has been assayed as a physiological mechanism which is impaired at early stages in AD. In this sense, a large set of related biomarkers were determined in plasma samples, selecting two compounds in the development of an AD differential diagnosis model. The corresponding internal validation was satisfactory, and further external validation of the developed model will be carried out as a fundamental stage before being applied in the clinical routine use. This is a promising screening test that could avoid the current invasive diagnosis method and could be useful in diagnosis and investigation.

## Figures and Tables

**Table 2:** Clinical and demographic variables for the participants.

Variables	AD Group (*n* = 138)	Healthy Group (*n* = 50)	Non-AD Group (*n* = 70)
Age (years, median (IQR))	71 (68, 74)	67 (62, 69)	66 (62, 71)
Gender (female, *n* (%))	80 (59.7%)	19 (38.78%)	31 (48.44%)
RBANS.DM (median (IQR))	44 (40, 56)	100 (92, 106)	64 (52, 81)
CDR (median (IQR))	0.5 (0.5–1)	0 (0–0)	0.5 (0.5–1)
β-amyloid (pg mL^−1^, median (IQR))	580 (464, 694)	1085 (924, 1308)	1049 (850, 1264)
t-Tau (pg mL^−1^, median (IQR))	707 (428, 830)	255 (144, 313)	322 (190, 395)
p-Tau (pg mL^−1^, median (IQR))	99 (71, 110)	47 (32, 60)	52 (34, 61)

**Table 3:** Analytes concentrations in plasma samples from participants groups.

Variable Median (IQR) (nmol L^−1^)	AD Group (*n* = 138)	Healthy Group (*n* = 50)	Non-AD Group (*n* = 70)	*P*-Value (Kruskal–Wallis)
	**Median (IQR)**	**Median (IQR)**	**Median (IQR)**	
15(*R*)-15-F_2t_-IsoP	0.21 (0.12, 0.32)	0.19 (0.13, 0.29)	0.19 (0.09, 0.33)	0.361
PGE_2_	0.08 (0, 0.38)	0.08 (0.02, 0.36)	0.12 (0.03, 0.36)	0.913
2,3-dinor-iPF_2__α_-III	0 (0, 0)	0 (0, 0)	0 (0, 0)	0.418
15-keto-15-E_2t_-IsoP	0.04 (0, 0.13)	0.03 (0, 0.14)	0 (0, 0.2)	0.924
15-keto-15-F_2t_-IsoP	0.14 (0.06, 0.37)	0.14 (0.09, 0.23)	0.16 (0.1, 0.33)	0.872
15-E_2t_-IsoP	0.2 (0.09, 0.93)	0.2 (0.12, 0.64)	0.48 (0.18, 1.05)	0.041 *
5-F_2t_-IsoP	0.77 (0.37, 1.45)	1.12 (0.54, 1.46)	1.08 (0.45, 1.55)	0.542
15-F_2t_-IsoP	0.03 (0.01, 0.06)	0.02 (0.01, 0.04)	0.01 (0, 0.07)	0.129
PGF_2α_	0.43 (0.17, 0.91)	0.78 (0.4, 1.08)	0.62 (0.3, 1.13)	0.005 *
4(*RS*)-F_4t_-NeuroP	1.2 (0.59, 1.44)	1.22 (0.7, 1.43)	0.5 (0, 1.43)	0.006 *
1a,1b-dihomo-PGF_2α_	0 (0, 0)	0 (0, 0)	0 (0, 0)	0.178
10-*epi*-10-F_4t_-NeuroP	0.13 (0.05, 0.2)	0.13 (0.07, 0.18)	0.22 (0.17, 0.31)	<0.001 *
14(*RS*)-14-F_4t_-NeuroP	0.56 (0.1, 1.2)	0.62 (0, 1.33)	0.52 (0.1, 1.48)	0.891
IsoP^$^	0.36 (0.26, 0.55)	0.31 (0.19, 0.45)	0.54 (0.42, 0.93)	<0.001 *
Ent-7(*RS*)-F_2t_-dihomo-IsoP	0.12 (0.08, 0.17)	0.11 (0.07, 0.15)	0.13 (0, 0.45)	0.181
17-F_2t_-dihomo-IsoP	0 (0, 0)	0 (0, 0)	0 (0, 0)	0.989
17-*epi*-17-F_2t_-dihomo-IsoP	0 (0, 0.02)	0 (0, 0)	0 (0, 0.18)	0.168
17(*RS*)-10-*epi*-SC-Δ^15^-11-dihomo-IsoF	0 (0, 0)	0 (0, 0)	0 (0, 0)	0.536
7(*RS*)-ST-Δ^8^-11-dihomo-IsoF	0.06 (0, 0.12)	0.11 (0, 0.18)	0.02 (0, 0.1)	0.155
NeuroF^$^	0.13 (0.06, 0.25)	0.07 (−0.1, 0.25)	0.14 (0.08, 0.2)	0.022 *
IsoF^$^	0.14 (0.08, 0.29)	0.11 (0.07, 0.3)	0.2 (0.08, 0.39)	0.336

^$^ Arbitrary units: (intensity of signal units × (internal standard concentration, nmol L^-1^); * *P* < 0.05; IQR: Inter-quartile range.
